# Health Disparities in COVID-19: Implications for Research, Policy, and Practice

**DOI:** 10.1093/geroni/igab046.748

**Published:** 2021-12-17

**Authors:** Chivon Mingo, Ronica Rooks

## Abstract

The rapid transmission of COVID-19 has resulted in more than 100 million confirmed cases in over 200 countries and continues to have wide-community spread. Consistently, it has been reported that older adults are at a greater risk for requiring hospitalization or dying from the virus compared to younger adults and children. In fact, compared to those age 18-29, age 65-74 are five times more likely to be hospitalized and 90 times more likely to experience death. The risk increases exponentially with age. Individuals 85 and older are 13 times more likely to require hospitalization and 630 times more likely to die from the disease. The physical health-age correlation has permeated the media and many discussions concerning the pandemic. However, fewer discussions have centered on the interaction of age and social variables that further exacerbate COVID-19 related burden or mortality such as race/ethnicity, socioeconomic status, and limited access to healthcare. Therefore, this symposium will bring direct attention to COVID-19 related health disparities that compromise public health, discuss implications on future research, policy, and practice, and discuss opportunities to reduce the burden and mitigate health inequities. The symposium presenters will specifically address the impact of social support during COVID-19, disparities in the effects of social distancing on health status, the economic impact on health, cognitive decline among low-income older adults navigating a global pandemic, and factors associated with higher rates of hospitalizations among racial/ethnic diverse older adults.

